# Synthetic dataset for visco-acoustic imaging

**DOI:** 10.1016/j.dib.2023.109199

**Published:** 2023-05-03

**Authors:** Florian Faucher, Otmar Scherzer

**Affiliations:** a Faculty of Mathematics, University of Vienna, Oskar-Morgenstern-Platz 1, A-1090 Vienna, Austria; b Team Makutu, Inria Bordeaux, TotalEnergies, Université de Pau et des Pays de l'Adour, CNRS, UMR 5142 France; c Johann Radon Institute for Computational and Applied Mathematics (RICAM), Altenbergerstraße 69, A-4040 Linz, Austria; d Christian Doppler Laboratory for Mathematical Modeling and Simulation of Next Generations of Ultrasound Devices (MaMSi), Oskar-Morgenstern-Platz 1, A-1090 Vienna, Austria

**Keywords:** Inverse problems, Visco-acoustic models, Time-harmonic wave propagation, Full waveform inversion

## Abstract

We provide computationally generated dataset simulating propagation of ultrasonic waves in viscous tissues in two and three dimensional domains. The dataset contains physical parameters of a human breast with a high-contrast inclusion, the acquisition setup with positions of sources and receivers, and the associated pressure-wave data at ultrasonic frequencies. We simulated the wave propagation based on seven different viscous models using the physical parameters of the breast. Furthermore, different choices of conditions for the medium's boundaries are given, namely absorbing and reflecting boundaries. The dataset allows to evaluate the performance of reconstruction methods for ultrasound imaging under attenuation model uncertainty, that is, when the precise attenuation law that characterizes the medium is unknown. In addition, the dataset enables to evaluate the robustness of inverse scheme in the context of reflecting boundary conditions where multiple reflections illuminate the sample, and/or the performance of data-processing to suppress these multiple reflections.

**Specifications Table**

**Table:** 

Subject	Mathematical modeling – Computational Mathematics
Specific subject area	Viscous materials - ultrasonic wave imaging - inverse problems - visco-acoustic wave propagation
Type of data	Models of physical properties of breast sample – Pressure wave data – 2D and 3D synthetic data
How the data were acquired	Synthetic data of visco-acoustic wave propagation are generated using software hawen, [7] (https://ffaucher.gitlab.io/hawen-website). The geometry of the structures for the samples are obtained from a cross-section of the OABreast Phantom dataset [11] (https://anastasiolab.wustl.edu/downloadable-content/oa-breast-database). The values of the physical properties in each layer of the sample is selected according to the IT'IS Database (https://itis.swiss/virtual-population/tissue-properties).
Data format	Raw
Description of data collection	Data are computationally generated
Data source location	Faculty of Mathematics, University of Vienna, Oskar-Morgenstern-Platz 1, A-1090 Vienna, Austria.
Data accessibility	The data are on the University of Vienna repository phaidra: https://phaidra.univie.ac.at/o:1637853, doi:11353/10.1622130, as well as on the Mendeley repository: https://data.mendeley.com/datasets/jh63d6jmmc/2, doi:10.17632/jh63d6jmmc.1.
Related research article	F. Faucher and O. Scherzer, *Quantitative inverse problem in visco-acoustic media under attenuation model uncertainty*, Journal of Computational Physics, 472 (2023) 111685. https://doi.org/10.1016/j.jcp.2022.111685.

## Value of the Data

• The dataset provides simulations of the pressure fields showing the propagation of ultrasonic waves in a breast sample for seven models of attenuation. This is the first collection of such simulations. It is therefore useful for visualization effects of different viscosity models on the propagation of waves. In addition, these data can be used for evaluation of computational ultrasound imaging (inversion) techniques. In particular, two medium configurations for the data are provided: An implementation with absorbing conditions that approximate the free-space propagation which is very often used in the physics literature, and a configuration with reflecting (wall) conditions.
• The dataset offers two and three dimensional benchmark data for visco-acoustic imaging that can be used by researchers and engineers working on inverse wave problems.
• The data can be used by experimentalists and computational scientists as a basis for testing computational ultrasound inversion techniques under several emitter and receiver configurations, as well as for different attenuation models characterizing the propagation within the sample.

## 1 Objective

The dataset made available for the community can be used for the following purposes:

1. For the comparison of computationally simulated wave propagation in viscous media: We provide data for different viscous models and different variants of implementations, in particular free-space and implementation on compact sets.
2. For ultrasound inversion algorithms based on (partial) data and for investigating reconstructions with different models of attenuation.
3. For the investigation of the scalability of simulations (forward model) and ultrasound inversion algorithms as we provide simulations of wave propagation in viscous media in space dimensions two and three.

In the related research paper [9], the dataset allows to develop and validate an efficient methodology for the reconstruction of visco-acoustic media with attenuation model uncertainty, and with different choices of boundary conditions surrounding the sample.

## 2 Data Description

The folder hierarchy separates the two and three-dimensional datasets:

• Folder 2D/ contains simulated data of two-dimensional wave propagation and the associated material parameters of the 2D sample.
• Folder 3D/ contains simulated data of three-dimensional wave propagation and the associated material parameters of the 3D sample.

Each folder further contains three subfolders which are detailed in the below table for the 2D numerical experiment, and the 3D experiment uses the same organization and structure of files, however with less choices of attenuation models. We further refer to Table 1 for more details.

**Table 1:** Directories for the 2D dataset.

2D dataset folder	
*2D/acquisition*	It contains two text files: *sources.txt* and *receivers.txt* that correspond to the list of the 2D positions of the sources and of the receivers. The first line of each file indicates the number of sources (36) and of receivers (360), then each subsequent line gives the index of the source/receiver and the two coordinates of its position, illustrated in Figs. 1 and 2.

**Fig. 1:**
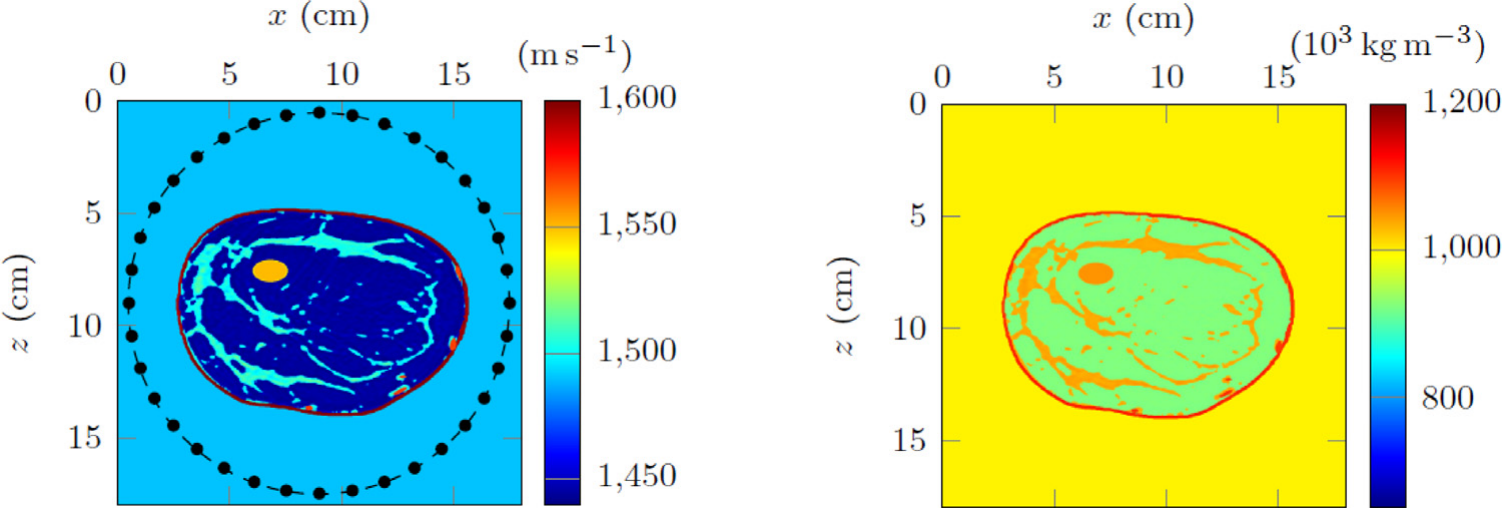
Configuration of the two-dimensional experiment which is of size 18 × 18cm^2^, with the wave speed (left subfigure) that corresponds to binary file 2D/models/main/cp_1001×1001.bin and density (right subfigure) to file 2D/models/main/rho_1001×1001.bin. The models of viscosity have the same structures, however with different scales adapted to each attenuation models (Table 2); these scales are chosen such that all attenuation models coincide at frequency 300 kHz, [9]. The positions of the sources are indicated with • and the receivers are along the dashed line. These positions are listed in files 2D/acquisition/sources.txt and 2D/acquisition/receivers.txt respectively.

*2D/models* *2D/models/main* *2D/models/attenuation*	It contains the stored parameters of the breast phantom. Each parameter is described by two files: a header (extension *.H*) that indicates the discretization step and sizes of the associated binary file (extension *.bin*) that contains the values of the physical parameter. Each binary consists of a 1001 × 1001 array of real numbers which correspond to the pixel representation of the parameter.It contains the wave speed (*cp_1001 × 1001*), density (*rho_1001 × 1001*) and quality factor at 300kHz (*Q_300kHz_1001 × 1001*). The wave speed and density are further pictured Fig. 1.In the other subfolders are the material parameters depending on the choice of attenuation models, with seven options, cf. [9]. For instance, folder *kelvin-voigt/* contains the physical parameters to simulate the Kelvin–Voigt attenuation model within the medium.

*2D/wave-dataset/*	It contains simulated pressure fields for various visco-acoustic models. In the first subdirectory, there are two subfolders, each contains simulated pressure fields for a pair of attenuation models and boundary conditions. The data are saved in text files, with one file per source and per frequency: For instance, file *data-record_p_frequency_0.00000E+00_2.00000E+05Hz_src000010.txt* corresponds to simulation at 200kHz (indicated by *2.00000E+05Hz*), for the source number 10 (indicated by *src000010*).Moreover, the first number corresponds to the imaginary part of the frequency, see Eq. (1): This is only non-zero in the case of wall boundary conditions, and we specify below the computed frequencies.Each file contains 360 complex values that correspond to the pressure value at the positions of the receivers. Note that the positions of the sources and receivers can be found in the acquisition folder described above.In Fig. 3, we illustrate graphically one of this file with the real and imaginary parts of the provided pressure field.

**Model description.** For the two-dimensional experiment, the physical parameters are given on a Cartesian grid (folder *2D/models/*) of size 1001 × 1001 with a discretization step of 0.18mm. That is, we use models of size 18 × 18cm^2^. The three-dimensional physical parameters are given on a Cartesian grid of size 121 × 191 × 201 with discretization steps of 0.833mm along x-axis, 0.842mm along y-axis and 0.800mm along z-axis. Therefore, the 3D models are of size 10 × 16 × 16cm^3^.

**Frequency content of the data.** We consider complex frequencies according to Eq. (1). For the two-dimensional experiment, the frequencies simulated are the following:

• With absorbing boundary conditions, we provide the pressure waves for real frequencies 200, 300, 400, 500 and 600 kHz.
• With wall boundary conditions, the pressure field are provided for the real frequencies 200, 300, 400, 500 and 600 kHz, and the same ones including imaginary part 10 × 10^3^, 15 × 10^3^ and 20 × 10^3^.

The frequencies simulated for the three-dimensional experiment are the following:

• With absorbing boundary conditions, we provide frequencies 100, 200, 300 and 400 kHz.
• With wall boundary conditions, we provide the ordinary frequencies 100, 200, 300 and 400 kHz, and the same frequencies including imaginary part: 10 × 10^3^, 15 × 10^3^ and 50 × 10^3^.

**Fig. 2:**
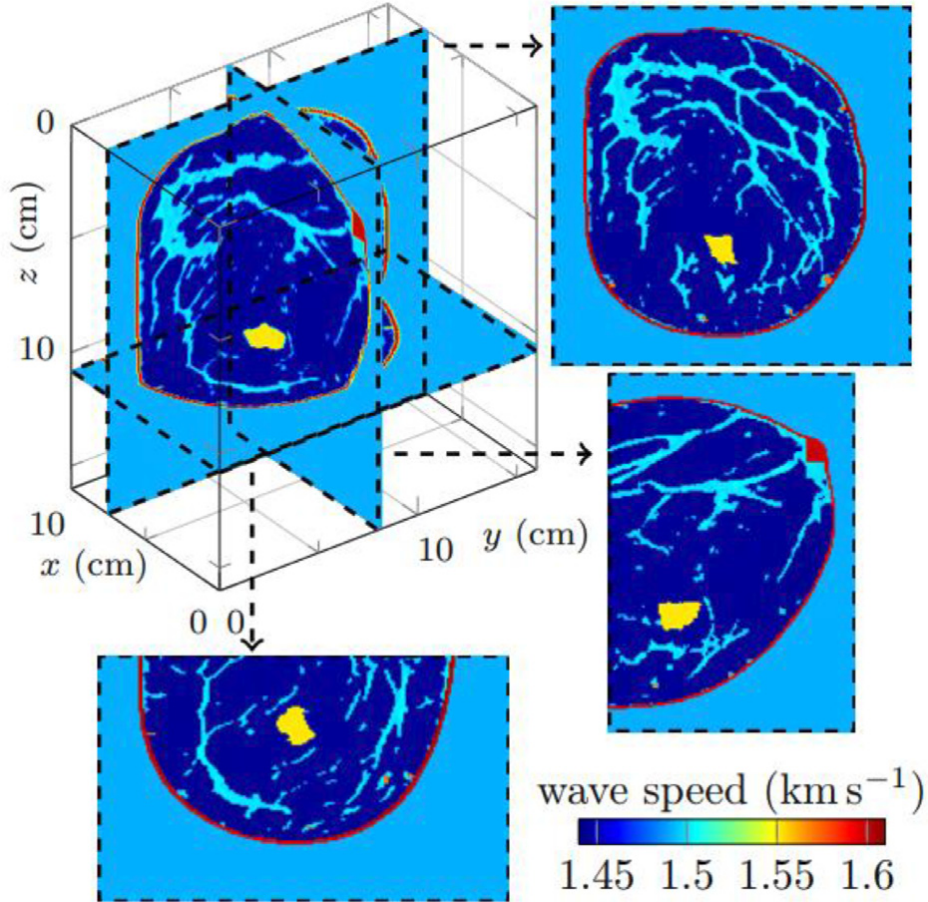
Three-dimensional wave speed model of size 10 × 16 × 16cm^3^ that corresponds to binary file 3D/models/main/cp_121 × 191 × 201.bin. We picture the cross-sections corresponding to y-z plane (top right), x-z plane (bottom right) and x-y plane (bottom left). In the numerical simulations with wall conditions, an absorbing boundary condition is imposed on the y-z plane for x=x_max_=10cm, to mimic the body.

**Fig. 3:**
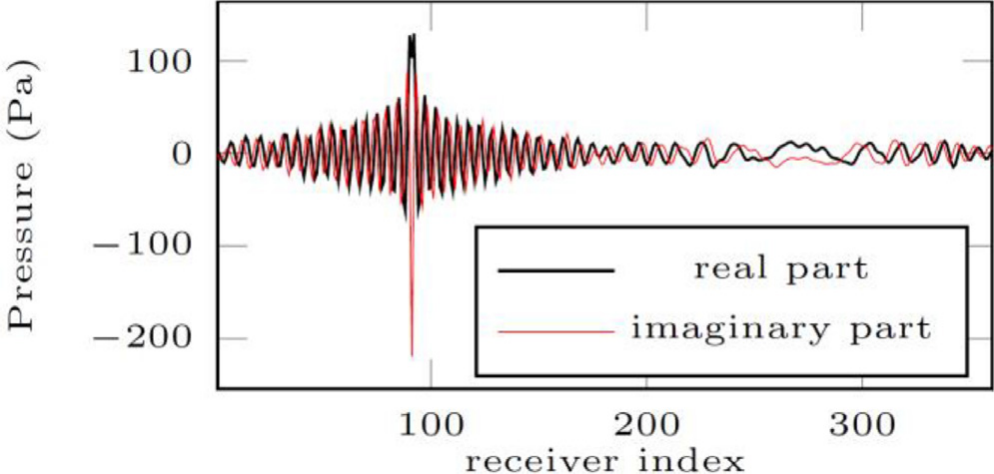
Pressure field saved for the 2D case at frequency 200kHz for the source numbered 10, in the configuration of absorbing boundary conditions with Kelvin–Voigt model of attenuation. It corresponds to file 2D/wave-dataset/data_absorbing-boundary-conditions/kelvin-voigt/receivers/data-record_p_frequency_0.00000E+00_2.00000E+05Hz_src000010.txt.

## 3 Experimental Design, Materials and Methods

The objective of inversion is to reconstruct the structures of the medium from wave measurements: these configurations arise in applications such as medical imaging [5,10], seismic imaging [14], and non-destructive testing. The dataset we provide consists in simulated pressure waves that propagate through a breast sample and that are recorded at receivers positioned outside the sample, as illustrated in Fig. 1. One difficulty of visco-acoustic media is that several mathematical models of attenuation exist, [2,13], and the precise model that characterizes the medium of interest is usually unknown a priori. This increases the difficulty of the reconstruction.

Attenuation is frequency-dependent such that time-harmonic formulation of wave equation is more appropriate [1,12] for discretization. Indeed, time-domain equations with attenuation can result in integro-differentiable equations while the frequency domain can be described by partial differential equations and complex-valued parameters, [13,3]. In our work, we further allow for *complex frequency* [8,9] such that

(1) $$\omega =2\pi \nu +i\;\omega_{i},$$

where ν is the ordinary frequency and $\omega_{i}$ is the imaginary part of the frequency. The propagation is given by the pressure field p and particle velocity v that solve the system of equations:

(2a) −iωρ(x)v(x,ω)+∇p(x,ω)=0inΩ,

(2b) $$-\frac{\;i\omega }{\;\kappa_{†\;}\left(x,\omega \right)}\;p\left(x,\omega \right)+\;\nabla \cdot v\left(x,\omega \right)=\;\delta \left(x_{s}\right)\;\text{in}\;\Omega .$$

Two alternatives are considered for boundary conditions defined on ∂Ω. Denoting n the normal direction, we have:

$$\begin{array}{c} \text{absorbing}\;\text{boundary}\;\text{conditions}:\;-{\left(\rho \;\kappa_{†\;}\right)}^{-1/2}\;p+\;v\cdot n\;=\;0\\ \text{reflecting}\;\text{boundary}\;\text{conditions}:\;\nabla p\cdot n=0 \end{array}$$

Here $x_{s}$ is the position of the source which is taken as a Dirac function in x. The density is ρ and the complex bulk modulus $\kappa_{†\;}$ depends on the choice of attenuation model and is expressed in terms of a real-valued bulk modulus $\kappa_{0}\left(x\right)=c_{0}{\left(x\right)}^{2}\rho \left(x\right)$ where $c_{0}$ is the wave speed (Fig. 1).

In order to solve the wave Equation (2) in the different configurations to generate the synthetic data, we use the software hawen, cf. [7]. It relies on the hybridizable discontinuous Galerkin discretization method [4], with precise implementation described in [8]. Consequently, the data generation follows those steps:

1. Using software hawen, find (p,v) that solve the wave Equation (2) for given
  a. domain Ω (2D or 3D);
  b. boundary conditions for the domain (absorbing or reflecting);
  c. parameters ρ and $\kappa_{†\;}$.
  d. source position;
  e. frequency ω.
2. Extract the value of the pressure field p solution to the wave equation at the positions of the receivers (illustrated in Fig. 1 for 2D).

As indicated above, the 2D domain is of size 18 × 18cm^2^ and in 3D, the domain is of size 10 × 16 × 16cm^3^. For the numerical resolution, the input parameters are given on a Cartesian grid (see the model description above). However, the computations are performed on an unstructured mesh. The parameters (the bulk modulus $\kappa_{†\;}$ and density ρ) are first represented with respect to the mesh via Lagrange basis functions to ensure the accurate representation (see Fig. 7 in [8]). The solutions are represented using a piecewise-polynomial representation such that the values of the pressure field are saved at the exact position of the receivers (without need for interpolation).

The formulation of $\kappa_{†\;}$ depending on the choice of attenuation model is given in the below table. The different models are extracted from [13,^2^,6] see also [9] and the references therein.

In Table 3, we summarize the main characteristics of the dataset for the two and three-dimensional cases.

**Table 2:** List of complex bulk modulus depending on the attenuation model.

Attenuation model	Complex bulk modulus formula
Simplified Kolsky-Futterman	$\kappa_{†\;}^{\left(kf\right)}=\;\kappa_{0}-i\;\frac{\kappa_{0}}{\eta }$
Cole-Cole	$\kappa_{†\;}^{\left(cc\right)}=\;\kappa_{0}\;\frac{1+{\left(-i\omega \tau_{\epsilon }\right)}^{\beta }}{1+{\left(-i\omega \tau_{\sigma }\right)}^{\beta }},\;\text{with}\;\beta =0.8$
Zener	$\kappa_{†\;}^{\left(z\right)}=\;\kappa_{0}\;\frac{1-i\omega \tau_{\epsilon }}{1-i\omega \tau_{\sigma }}$
Kelvin-Voigt	$\kappa_{†\;}^{\left(kv\right)}=\;\kappa_{0}-i\;\omega \kappa_{0}\tau_{\epsilon }$
Maxwell	$\kappa_{†\;}^{\left(m\right)}=\;\frac{-\;i\;\omega \kappa_{0}\eta }{\kappa_{0}-\;i\omega \eta }$
KSB (Kowar-Scherzer-Bonnefond)	$\kappa_{†\;}^{\left(ksb\right)}=\;\kappa_{0}\;\frac{\kappa_{0}}{{\left(1+\;\frac{\eta }{\sqrt{1+{\left(-i\omega \tau \right)}^{\beta }}}\right)}^{2}},\;\text{with}\;\beta =0.5$
Modified Szabo	$\kappa_{†\;}^{\left(sz\right)}=\;\frac{\kappa_{0}}{1+{\left(-i\omega \tau \right)}^{\beta -1}},\;\text{with}\;\beta =0.5$

**Table 3:** Summary of the 2D and 3D wave dataset.

2D experiment	
**Domain size**	18 × 18cm^2^
**Number of independent sources**	36
**Number of receivers per source**	360
**Representation of the model parameters**	Cartesian grid with step 0.018cm in both directions
**Frequency content in the data**	
• with absorbing boundary conditions.	Ordinary frequency: 200, 300, 400, 500 and 600 kHz, Imaginary part: 0.
• with wall boundary conditions.	Ordinary frequency: 200, 300, 400, 500 and 600 kHz, Imaginary part: 0, 10 × 10^3^, 15 × 10^3^ and 20 × 10^3^.

3D experiment	

**Domain size**	10 × 16 × 16cm^3^
**Number of independent sources**	224
**Number of receivers per source**	1533
**Representation of the model parameters**	Cartesian grid with step 0.833mm along x-axis, 0.842mm along y-axis and 0.800mm along z-axis
**Frequency content in the data**	
• with absorbing boundary conditions.	Ordinary frequency: 100, 200, 300 and 400 kHz, Imaginary part: 0.
• with wall boundary conditions.	Ordinary frequency: 100, 200, 300 and 400 kHz, Imaginary part: 0, 10 × 10^3^, 15 × 10^3^ and 50 × 10^3^.

## Ethics Statements

This work meets the ethical requirements of the Journal Data in Brief. Furthermore, this work does not involve experiments on humans or animals.

## CRediT authorship contribution statement

**Florian Faucher:** Conceptualization, Methodology, Software, Validation, Formal analysis, Investigation, Resources, Data curation, Writing – original draft, Writing – review & editing, Visualization, Project administration, Funding acquisition. **Otmar Scherzer:** Conceptualization, Formal analysis, Investigation, Resources, Writing – review & editing, Project administration, Supervision, Funding acquisition.

## Declaration of Competing Interest

The authors declare that they have no known competing financial interests or personal relationships that could have appeared to influence the work reported in this paper.

## Data Availability

Synthetic dataset for visco-acoustic imaging (Original data) (phaidra). Synthetic dataset for visco-acoustic imaging (Original data) (Mendeley Data).
